# An Early Gestation Plasma Inflammasome in Rural Bangladeshi Women

**DOI:** 10.3390/biom14070736

**Published:** 2024-06-21

**Authors:** Hyunju Kim, Jacquelyn R. Bedsaul-Fryer, Kerry J. Schulze, Gwen Sincerbeaux, Sarah Baker, Casey M. Rebholz, Lee SF Wu, Joseph Gogain, Lena Cuddeback, James D. Yager, Luigi M. De Luca, Towfida J. Siddiqua, Keith P. West

**Affiliations:** 1 Department of Epidemiology, Johns Hopkins Bloomberg School of Public Health, Baltimore, MD 21205, USA; 2 Department of Epidemiology, School of Public Health, University of Washington, Seattle, WA 98105, USA; 3 Cancer Prevention Fellowship Program, Division of Cancer Prevention, National Cancer Institute, Rockville, MD 20850, USA; 4 Department of International Health (Human Nutrition), Johns Hopkins Bloomberg School of Public Health, Baltimore, MD 21205, USA; 5 Department of Nutritional Sciences, Pennsylvania State University, University Park, PA 16802, USA; 6 SomaLogic Inc., Boulder, CO 80301, USA; 7 Department of Environmental Health and Engineering, Johns Hopkins Bloomberg School of Public Health, Baltimore, MD 21205, USA; 8 The JiVitA Project, Rangpur 8240, Bangladesh

**Keywords:** proteomics, inflammation, pregnancy, biomarkers

## Abstract

Circulating α1-acid glycoprotein (AGP) and C-reactive protein (CRP) are commonly measured to assess inflammation, but these biomarkers fail to reveal the complex molecular biology of inflammation. We mined the maternal plasma proteome to detect proteins that covary with AGP and CRP. In 435 gravida predominantly in <12-week gestation, we correlated the relative quantification of plasma proteins assessed via a multiplexed aptamer assay (SOMAScan^®^) with AGP and CRP, quantified by immunoassay. We defined a plasma inflammasome as protein correlates meeting a false discovery rate <0.05. We examined potential pathways using principal component analysis. A total of 147 and 879 of 6431 detected plasma proteins correlated with AGP and CRP, respectively, of which 61 overlapped with both biomarkers. Positive correlates included serum amyloid, complement, interferon-induced, and immunoregulatory proteins. Negative correlates were micronutrient and lipid transporters and pregnancy-related anabolic proteins. The principal components (PCs) of AGP were dominated by negatively correlated anabolic proteins associated with gestational homeostasis, angiogenesis, and neurogenesis. The PCs of CRP were more diverse in function, reflecting cell surface and adhesion, embryogenic, and intracellular and extra-hepatic tissue leakage proteins. The plasma proteome of AGP or CRP reveals wide proteomic variation associated with early gestational inflammation, suggesting mechanisms and pathways that merit future research.

## 1. Introduction

Pregnancy is a dynamic, inflammation-regulated state that enables and protects the mother, placenta, and fetus to coexist, progressing through complex stages of implantation, growth, and development [1,2,3]. Early pregnancy is characterized by a slightly proinflammatory phase, accompanied by major maternal immune adaptations [4], when immune cells (macrophages, natural killer cells, and dendritic cells) infiltrate the decidua and surround the newly forming trophoblast cells to support placental development, implantation, and decidual formation [1]. The trophoblast sends signals to recruit immune cells toward the site of implantation, which is believed to switch immune cell differentiation toward an anti-inflammatory state in order to support fetal growth into the second trimester [1]. Despite immense advances in the knowledge of inflammation in reproductive biology [5], the exploration of the diversity, function, and potential public health application of the biomarkers of gestational inflammation, especially in low-resource settings, remains modest. 

Population-based epidemiologic studies have long restricted the assessment of inflammation to the measurement of two serum biomarkers, α-1 acid glycoprotein (AGP) as an indicator of chronic inflammation, and C-reactive protein (CRP) as a marker of acute inflammation [6,7,8], frequently using them to adjust other health or nutrition indicators (e.g., micronutrient status) for modulating the effects of presumed infection, despite the unclear functional roles of either in immune surveillance or response [9,10,11]. Deploying only two biomarkers also masks the immense complexity of inflammation and contributions of numerous proteins functioning locally and systemically in innate and adaptive immune systems [12,13]. Further, AGP and CRP may act in similar or opposing directions, rising and falling in plasma on different timelines and reflecting different multisystem sequences and levels of response to stress from infection, chronic disease, environmental exposure, or other metabolic or normal physiologic stress, including pregnancy [7,14,15,16]. 

Plasma proteomics embodies an array of high-throughput methodologies capable of detecting and quantifying an abundance of thousands of circulating proteins in a small sample of plasma [17]. Increasingly deployed to identify potentially predictive biomarkers of disease [18,19], nutritional status [20], and inflammation in children [21], plasma proteomics has been explored as a means to reveal physiological [22,23] and pathological biomarkers in pregnancy [23]. Previously, we used tandem mass spectrometry to detect 100 and 90 plasma proteins associated with AGP and CRP, respectively, at a false discovery rate (q) of < 0.10 in Nepalese school-aged children, proposing the term *plasma inflammasome* [21]. Included in these clusters were proteins involved in homeostatic and induced host defense, nutrient metabolism, and tissue repair, revealing an array of potential biomarkers with which to assess and differentiate the types of inflammation in populations [21]. 

In this study, we extend the exploration and quantification of a plasma inflammasome, linked to global inflammatory biomarkers of AGP and CRP, in a population sample of rural Bangladeshi women who were predominantly in the first trimester of pregnancy. Employing a high-throughput modified aptamer capture methodology [17,18], we characterize a vast *plasma inflammasome* comprising homeostatic, anabolic, catabolic, proinflammatory, and resolving biomarkers that, with further research, may lead to panels of indicators of early gestational inflammation. 

## 2. Materials and Methods

### 2.1. JiVitA-3 Trial

We used archived biospecimens of participants in the JiVitA-3 study, a cluster-randomized, double-masked trial conducted in the Gaibandha District of northern Bangladesh from 2008 to 2012 that examined the prophylactic efficacy of daily antenatal multiple micronutrient (MM) versus iron–folic acid (IFA) supplementation in reducing adverse pregnancy outcomes. Details on the study design, population, and effects on pregnancy outcomes have been reported previously [24]. Briefly, this location was selected because it typifies rural life in Bangladesh with respect to the extent of malnutrition, agriculture, infrastructure, health care, diet, and demographic factors [25]. 

Married, nonpregnant women aged 12–45 years living in 596 sectors (clusters of 25–400 households) across 19 rural unions were enlisted into a home-based pregnancy surveillance system. Trained data collectors visited a total of >127,000 listed women every 5 weeks to detect new pregnancies by eliciting a history of amenorrhea in the previous month and pregnancy confirmation by urine testing. A total of 44,567 consenting pregnant women were enrolled into the study at a median of 9 (interquartile range: 7–12, 5 weeks) weeks’ gestation. Participants underwent a baseline interview about the previous week’s diet, frequencies of work-related activities, presence of 23 morbidity symptoms (including nausea, vomiting, severe headache, high and low fever, diarrhea) conditioned on a positive history of ≥1 symptomatic days in the previous month, and social, educational, and ownership variables that were weighted and modeled into living standards and wealth indices [26]. Trained staff measured participants’ height, weight, and arm circumference. Further trial procedures, including 3rd trimester, birth, and post-partum assessments of mothers and infants have been reported elsewhere [25]. The current study was restricted to biospecimens from the trial’s baseline assessment. 

This proteomics study focused on 435 women with complete demographic, epidemiological, socioeconomic, biochemical, and other assessment data from the trial. Written informed consent was obtained from all participants. The JiVitA-3 trial was approved by the Bangladesh Medical Research Council, approved annually by the Institutional Review Board at Johns Hopkins Bloomberg School of Public Health (BSPH, IRB number: IRB00000570) on 10 December 2007, with latest annual approval on 27 October 2023. A Data and Safety Monitoring Board regularly followed the trial which was registered with ClinicalTrials.gov (NCT00860470). Data from the present study will not be shared publicly and will follow the parent study’s (JiVitA trial) data management and sharing procedures.

### 2.2. Biochemical Substudy 

A total of 64 contiguous sectors (approximately 10% of all sectors), centrally located in the trial area and balanced on supplement allocation, comprised a substudy area in which pregnant women identified during surveillance were invited to receive additional anthropometric (triceps and subscapular skinfolds), bioelectrical impedance, clinical (including blood pressure), and biochemical assessments [27]. Fasting blood specimens of 5–6 mL were collected from 2070 subjects at home in sodium-heparin-containing evacuated tubes, transported to a field laboratory in opaque coolers, centrifuged to plasma, aliquoted, and stored and shipped under liquid nitrogen to BSPH. Biospecimens were stored at −80 °C until analysis. At BSPH, α_1_-acid glycoprotein (AGP) and C-reactive protein (CRP) were measured using a radial immunodiffusion kit (Kent Laboratories, Bellingham, WA, USA) and a clinical chemistry analyzer (Immulite 1000, Siemens Diagnostics, Munich, Germany), respectively. The laboratory coefficients of variation (CVs) for AGP were 10.0% and 5.8% for CRP. Laboratory technicians were not aware of the intervention status. A cut-off of >1.0 g/L for AGP and >5.0 mg/L for CRP was used to identify individuals with inflammation [27]. 

### 2.3. Proteomics Substudy

A proteomics sampling frame of N = 1423 of a total of 2070 trial substudy plasma specimens in the JiVitA-3 bioarchive was constructed based on several criteria: having no incomplete or missing demographic, epidemiological, socioeconomic, biochemical, or other assessment data from the trial. The characteristics of the 1423 selected women without any missing data were highly comparable to the 747 women excluded for having any missing information. From this sampling frame, n = 450 women who were predominantly in their 1st trimester (baseline) plasma samples, 225 from each of the two trial supplement allocation groups were randomly selected for proteomics analysis. 

Aliquots of 150 µL for each of the 450 pregnant women, plus 20 randomly sampled, masked duplicates from the same group of women, were sent to SomaLogic (Boulder, CO, USA) where samples were analyzed by SomaScan^®^ (SomaLogic, Boulder, CO, USA), a highly sensitive aptamer-based proteomics assay which utilizes chemically modified nucleotides, called SOMAmer^®^ (Slow Off-rate Modified Aptamer) reagents that bind to proteins throughout the fM-to-µM range [17,28]. Through a previously described sequence of the capture, recapture, and washing process [17,28], SomaScan detected and quantified 7596 aptamer-bound protein targets, and the median coefficient of variation (CV) was approximately 5%. We excluded 233 protein targets that were bound to FC-mouse and 73 protein targets that the SomaScan assay identified as non-proteins (e.g., hybridization control elution aptamers or classified as non-human proteins). Of the remaining 7290 protein targets, 859 isomers were detected as duplicate proteins (i.e., >1 targets aligning with a given protein). Therefore, 6431 proteins had unique Entrez Gene and UniProt identifiers. Our analyses were based on the 7290 protein targets, and all were log_2_-transformed to reduce skewness. Masked duplicates generated from our study participants showed excellent SomaLogic assay reproducibility (median Pearson *r* = 0.92, median CV = 4.8%).

Among the original 450 specimens, 14 specimens flagged by the SomaScan assay as being of poor quality due to insufficient volume were excluded, and one specimen was not analyzed, leaving 435 specimens available for evaluating plasma proteins associated with AGP. An additional 15 specimens were excluded due to the lack of biochemically determined CRP data, leaving 420 specimens for exploring proteins associated with CRP. CRP was log_2_-transformed to improve the normality of the distribution.

### 2.4. Statistical Analysis

Following the examination of 1st trimester continuous and categorical characteristics, we employed simple linear regression models to obtain Pearson correlation coefficients (*r*) quantifying the linear relationship between 7290 protein targets and plasma AGP and CRP concentrations. A chance-corrected *p*-value [(FDR, q) [29] of <0.05] was adopted to define suites of plasma proteins associated with AGP and CRP, referred to as the *AGPome* and *CRPome*, respectively. To explain variances in the distributions of AGP and CRP, we performed principal component analysis (PCA) and explored linear combinations of proteins that were significantly associated with AGP and CRP. The first three principal components (PCs) that explained approximately 40% of the variation in AGP and CRP were selected. We also constructed correlation matrices with the top 40 proteins associated with AGP, based on q-values, to provide a view of the diversity of biological function and covariability. Lastly, we estimated the risks of having a positive 7-day history of maternal morbidity symptoms (nausea, vomiting, severe headache, high fever, and diarrhea) associated with each PC by estimating odds ratios (ORs) with associated 95% confidence intervals (CIs). All analyses were performed using R version 4.1.2.

## 3. Results

Table 1 displays comparability between the 435 women whose specimens were analyzed and 973 women whose samples were not included. Notably, 23% of women included in the current analysis had no formal schooling, one-third were <20 years, 30% were multiparous, 44% were underweight, and half were short in stature. The median gestational age at blood draw was 10 weeks (interquartile range: 8.0–12.3, 4.3 weeks). Approximately 20% and 11% had AGP and CRP concentrations > 1 g/L and 5 mg/L, respectively. 

Of the 7290 protein targets, the *AGPome* comprised 147 protein targets (2.3%) at q < 0.05 (Supplemental Tables S1 and S2). Fifty-five protein targets were positively correlated with AGP, including serum amyloid proteins (APCS, SAA1, SAA2, and SAA4), complement proteins (C1q, C1QC, CFB, CFI, and C9), interferon-induced proteins (IFIT3, MX1), immune signaling and regulatory proteins (LILRA1/5, IGSF3, and TIMD4), and CRP (q = 5.65 × 10^−6^). Of the top 50 proteins associated with AGP, major negative correlates included lipocalins (RBP4) and apolipoproteins (APOA1, APOC1, APOM), sex hormones (SHBG), and pregnancy-related proteins (CSH1, GPC3) (Table 2). 

Of 879 protein targets (13.7%) comprising the CRPome, 438 were positive correlates and 441 negative correlates, meeting a q < 0.05 (Supplemental Tables S3 and S4). CRP (SomaLogic assay) was strongly positively correlated with our biochemical indicator of CRP (r = 0.89; FDR value = 8.48 *×* 10^−144^) (Table 3). Of the top 50 proteins associated with CRP, most were also correlates of AGP [serum amyloid (APCS, SAA1/2), complement proteins (C1QC, CFB, CFI, C9), lipocalins (RBP4), and apolipoprotein (APOA5)]. 

**Figure 1:**
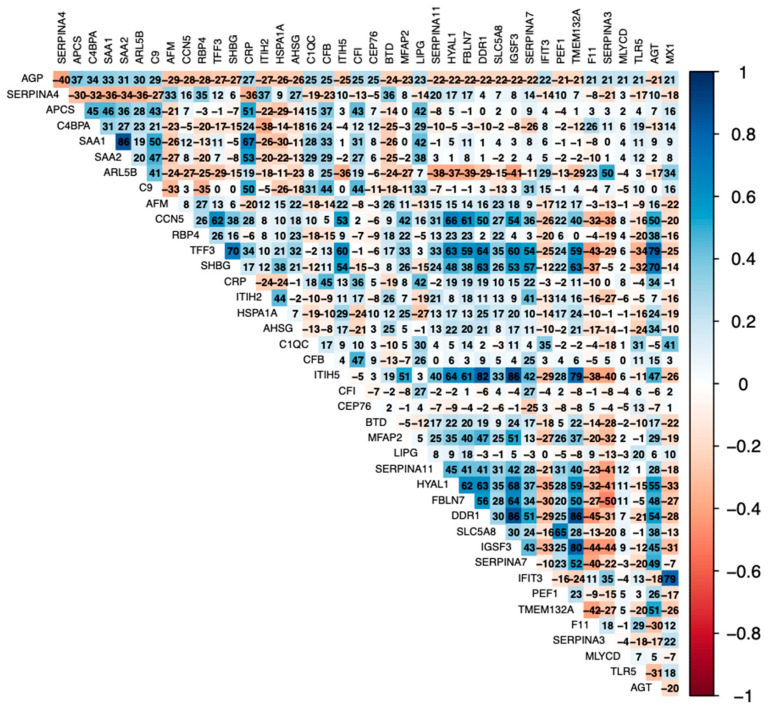
Matrix of correlation coefficients (*r*) for 40 proteins, measured in relative fluorescence units by SomaScan and presented as Entrez gene identifiers, most strongly associated with α_1_-acid glycoprotein (AGP), measured by radial immunodiffusion kit in g/L, listed in ascending order of chance-corrected *p*-values, with all q-values < 0.002 for displayed proteins. Blue color represents positive correlates, and red color represents negative correlates, with intensity of color reflecting extent of positive or negative correlations, respectively. Correlation coefficients are presented as *r* × 10^2^ to improve visualization. Full names and UniProt identifiers of proteins presented are shown in Table 2.

**Figure 2:**
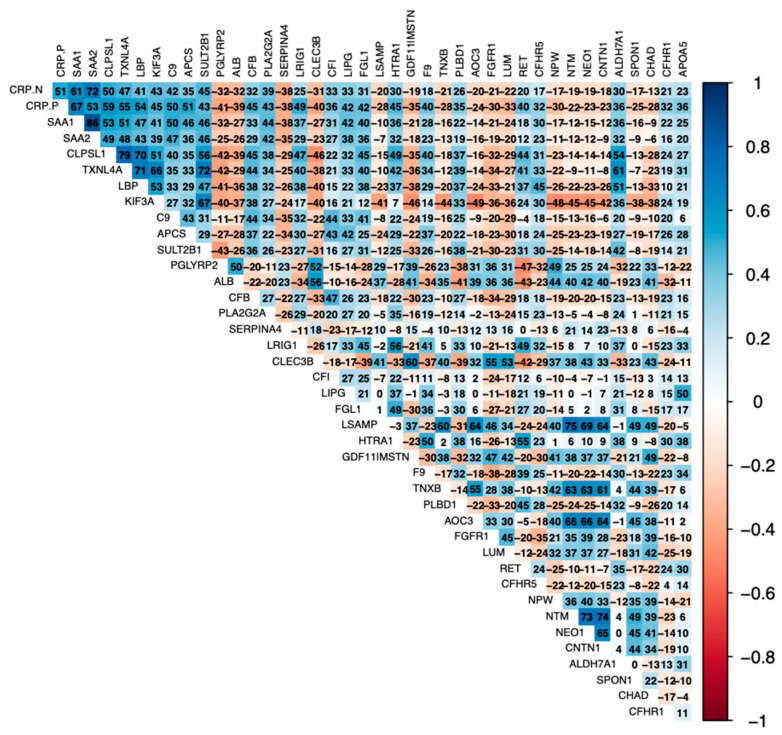
Matrix of correlation coefficients (*r*) for 40 proteins, measured in relative fluorescence units by SomaScan and presented as Entrez gene identifiers, most strongly associated with C-reactive protein (CRP), measured by clinical chemistry analyzer in mg/L, listed in ascending order of chance-corrected *p*-values (q), with all q-values < 3.00 × 10^−8^ for displayed protein pairs. Blue color represents positive correlates, and red color represents negative correlates, with intensity of color reflecting extent of positive or negative correlations, respectively. Correlation coefficients are presented as *r* × 10^2^ to improve visualization. Full names and UniProt identifiers of listed proteins shown in Table 3.

Positive correlates of AGP [complement proteins (C9, CFI, C1QC, CFB), serum amyloids (SAA1/2, APCS), and CRP] were positively correlated with each other (Figure 1). Further, IGSF3 was highly positively correlated with SERPINA4, SHBG, TFF3, and CCN5. Similarly, positive correlates of CRP were positively correlated with each other (serum amyloids, LBP, LIPG, complement proteins) (Figure 2). Negative correlates of CRP (APOC3, FGFR1, LUM, NPW, NTM, NEO1, CNTN1) were inversely related to proinflammatory serum amyloids, LBP, and complement proteins.

**Figure 3:**
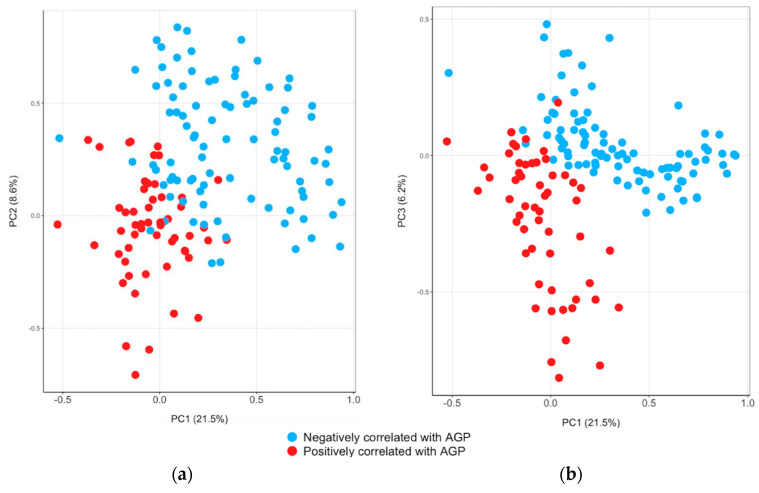
Bi-plots from principal component analysis. Principal component analysis was conducted among plasma proteins significantly associated with α-1acid glycoprotein (AGP) at Q values (FDR) < 0.05. (**a**) is a bi-plot of principal component 1 (PC1) and PC2. (**b**) is a bi-plot of PC1 and PC3. The percentages in parentheses on the axis indicate the proportion of the variance explained by each PC.

The PCA identified three components that explained 21.5%, 8.6%, and 6.2% of the variance in AGP (Supplemental Table S5; Figure 3). All proteins in the first component of AGP were negatively correlated with AGP, except for coagulation factor XI (F11). The first component of AGP had high positive loadings on CSH1 and PGF and negative loadings on ALB and F11. In the second component, the majority of proteins were negatively correlated with AGP. The second component of AGP had high loadings on B4GALT6, EGFLAM, and protogenin. The third component had high negative loadings on complement, serum amyloids (SAA1/2, APCS), and LBP and positive loadings on SERPINA4 and HSPA1A.

**Figure 4:**
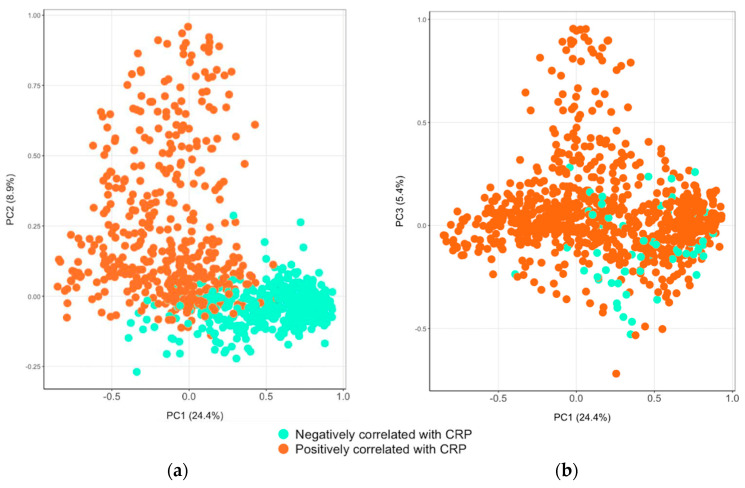
Bi-plots from principal component analysis. Principal component analysis was conducted among 879 plasma proteins significantly associated with C-reactive protein (CRP) at Q values (FDR) < 0.05. (**a**) is a bi-plot of principal component 1 (PC1) and PC2. (**b**) is a bi-plot of PC1 and PC3.

For CRP, the first to third components explained 24.4%, 8.9%, and 5.4% of the variance (Supplemental Table S6; Figure 4). The principal components of CRP were more diverse than the those of AGP. The majority of proteins with high positive loadings in the first component were dominated by cell surface, cell adhesion (neurotrimin, netrin receptors), and embryogenesis-related proteins. The second component of CRP had high loadings on intracellular and extra-hepatic tissue leakage proteins. The third component had high positive loadings in CSH1, glypican-3, pregnancy-specific beta-1-glycoprotein 11, and intestinal barrier proteins that support gastrointestinal immunity (e.g., TFF3) and other normal functions.

The PCs were associated with maternal morbidity symptoms (Table 4). The first (OR: 2.28, 95% CI: 1.59, 3.29), second (OR: 2.05, 95% CI: 1.37 3.11), and third (OR: 1.69, 95% CI: 1.08 to 2.77) scores of AGP per standard deviation were associated with higher odds of women reporting a high fever in the previous week. The PCs of AGP were associated with higher odds of nausea, vomiting, and severe headache. Similarly, the second or third PCs of CRP were associated with higher odds of vomiting, severe headache, or high fever.

## 4. Discussion

In this study of first-trimester gravida in Bangladesh, we identified a vast *plasma inflammasome* in early gestation, defined as proteins that covaried with the common inflammatory biomarkers AGP or CRP. The *AGPome* and *CRPome* proteins highlight the intricacy of pro- and anti-inflammatory and homeostatic processes to which these two biomarkers are linked. Among the inflammasome correlates are subsets of proteins associated with maternal morbidity, especially recent high fever, headache, and vomiting. It is possible that PCA provided proteins that are down- and up-regulated in maternal inflammation.

In this aptamer-based study, generating highly precise relative quantification and lacking missing data, we set q < 0.05, which accommodated an unknown breadth of early gestational inflammation. Alternatively, in an earlier mass spectrometric study in young Nepalese children, requiring a substantial imputation of missing values, we employed a more stringent threshold of q < 0.01 [21]. In both studies, there were similar but far from identical compositions of the plasma *AGPome* and *CRPome*. For example, in both life stages, positive correlates of AGP and CRP included complement proteins, protease inhibitors (SERPING1, SERPINA10), and serum amyloid A-1/A-2 proteins, all of which are expected to increase with inflammation [21]. In our study, negative correlates of AGP and CRP expectedly included transporters of micronutrients and lipids and anabolic hormone regulators. Among the negative correlates, RBP4 transports retinol, APOA-1 transports lipids, thyroxine-binding globulin (SERPINA7) transports thyroid hormones [30], and sex hormone-binding protein (SHBG) binds and mediates the bioavailability of sex steroids [31]. Many such proteins are characterized as anti-inflammatory [32,33,34,35]. In addition to these proteins, many observed correlates of AGP and/or CRP in this study might reflect complement-mediated apoptosis and the resultant release of cellular components into plasma, which naturally occur throughout pregnancy [36,37]. 

Our analysis identified a strong, negative correlation of AGP with plasma kallistatin, a circulating serine protease inhibitor with pleiotropic antioxidant, anti-inflammatory, and immunoregulatory functions affecting cardiovascular homeostasis [38]. A low plasma kallistatin level has recently been associated with an increased risk of chorioamnionitis in women experiencing preterm labor [39] and the preterm rupture of membranes [40], implicating its potential utility as a biomarker of chorionic inflammation. 

Lipopolysaccharide-binding protein (LBP), an acute-phase protein involved in eliciting an innate immune response, was positively correlated with AGP and CRP. LBP is secreted by hepatocytes into the serum and interacts with the Gram-negative bacterial lipopolysaccharide (LPS) with dual functions to neutralize proinflammatory responses against pathogens by promoting phagocytosis and the clearance of the pathogen and helping to bring the LPS antigen to its cognate receptor, CD14-toll-like receptor 4, on mononuclear target cells [41]. This latter interaction induces a proinflammatory innate immune response. Although it is not certain whether infection is present in the study population, given the presence of morbidity symptoms in early pregnancy, there are likely proinflammatory processes occurring [1,4,42]. 

Several proteins thought to function in intracellular immunity were correlated with AGP and CRP, suggesting processes of cellular death, protein degradation, and release into circulation. However, it may also indicate the extracellular systemic delivery of proteins, which may occur through the loading and transport of small extracellular vesicles (sEVs) [43,44]. Indeed, the interferon-induced protein with tetratricopeptide repeats 3 (IFIT3), a cytosolic protein with antiproliferative and antiviral properties that was positively correlated with AGP, has been previously located in sEVs and shown to exhibit a regulatory activity of protein expression in the vesicle and intercellular communication [44]. It is also possible that cell surface receptors (e.g., IL-22RA, the immunoglobulin receptor IGDCC4, TIMD-4, and CD74) that correlated with AGP and CRP in our study may have similar effector roles in sEVs. Any of these proteins may have physiological function in plasma, warranting further investigation.

The early gestation plasma inflammasome revealed paradoxical associations, perhaps unique to pregnancy. For example, chorionic somatomammotropin hormone 1/2 (CSH 1/2) and glypican-3 (GPC3) were negatively correlated with and received high loading for principal component 1 for AGP but were positively correlated with CRP. CSH1/2 is a part of the somatotropin and prolactin class of hormones, expressed in the placenta, whose expression rises early in gestation and remains high throughout pregnancy [45] as a peptide hormone enabling maternal–fetal nutrient transfer and fetal growth [46]. GPC3 is involved in regulating cellular growth, differentiation, and the regulation of growth factors involved in Wnt, hippo, and Hedgehog signaling pathways [47,48], possibly affecting morphogenesis, limb patterning, and skeletal development [49]. Plasma pregnancy-specific glycoprotein beta-1 (PSG1), a positive correlate of CRP secreted by the placenta, has been expressed in pregnant women from day 3 post-fertilization when the blastocyst implants to the uterine wall [50,51], a proinflammatory process requiring careful homeostatic control. PSGs have been observed to help establish the maternal–fetal vasculature [52]. Discordant associations observed between these proteins and AGP and CRP may reflect that AGP and CRP are differentially sensitivity to the timing of the inflammatory response. CRP responds more immediate than AGP; as CRP begins to resolve, AGP becomes elevated [16].

Complement surveillance is integral to innate immune responses to inflammation [53], which is critical for controlled utero-placental growth [36,37]. Many complement components were variably correlated with AGP and CRP. The complement C1q subcomponent subunit C, a key initiator of complement pathway activation, was positively correlated with both AGP and CRP [53,54]. Several early cascade proteins correlated with CRP but not AGP, including C2, a protease important early in the classical pathway, complement components C3 and C5 that mediate inflammation, and complement factor H-related proteins 1 and 5, which are inhibitory factors of the alternative pathway that rise in the first trimester [53]. Complement factor B (CFB), component 9 (C9), and factor I (CFI) were positively correlated with both AGP and CRP. CFB promotes the alternative complement activation pathway and has been shown to rise during the first trimester [53], while C9 is involved in downstream activation to form the membrane attack complex leading to the death of damaged cells. As a serum protease, activated CFI deattenuates complement activation through the degradation of certain complement proteins [55], thus protecting host tissues from inappropriate or prolonged complement activation. While seemingly paradoxical, a positive correlation of CFI with both CRP and AGP might illustrate an increased circulation of CFI as a zymogen [56], to be activated as needed to control inflammatory processes. These results appear to reflect immense heterogeneity in pro- and anti-inflammatory circuits underlying these two biomarkers.

Seeming paradoxical associations extended to other signaling molecules involved in immunoregulation. For example, the cytokine IL-10, a protein that acts within type 2 immunity to down-regulate inflammation [57], was negatively correlated with AGP. Similarly, the ubiquitin-like protein, ISG15, is an interferon-induced protein of the innate immune system that acts both intracellularly and extracellularly to regulate inflammatory responses. Its negative association with AGP, a more gradual and longer acting biomarker than CRP, may have been reflecting a chronic, low-level proinflammatory state [58].

The three examined PCs offered an ordered view of clusters of proteins explaining more to less variance in plasma concentrations of AGP and CRP, possibly reflecting the biological prominence of proteins involved in the down- and up-regulation of inflammation. The first and second components of AGP comprised predominantly negative correlates of AGP, anabolic agents linked to the maintenance of pregnancy (PGRMC2 [59]), embryogenesis (protogenin [60], DDR1 required for blastocyst implantation [61]), neurogenesis (TMEM132A [62], gliomedin [63]) including axon growth (semaphorin 6/7A [64]) and synapse formation (pikachurin [65]), and organ development (DDR1, fibulin-1 [66]). The third component of AGP included a broader mix of proinflammatory proteins, including those of the complement cascade, CRP, and serum amyloids. The composition of these sequential components may reflect priorities to tolerate the conceptus, promote embryo-feto-placental growth and development, and protect early pregnancy from inflammation while responding to infectious threats evident by the concurrent activation of acute-phase and catabolic proteins. The essentiality of the latter inflammatory mechanisms find support in principal components that were related to the symptoms of maternal morbidity, especially high fever. 

Our study has several strengths, including the use of large-scale proteomics, focus on pregnant women in low-resource settings, and rigorous ascertainment of biospecimens used for proteomics which reflect the source population. However, the present study used a cross-sectional design, making it challenging to infer causality due to the lack of temporality. Further, there was no information on the presence of infection in this population. Additionally, the present study did not investigate the association between significant proteins and pregnancy outcomes. Examining such associations is important but is beyond the scope of the present study. While the present study did not investigate the association between significant proteins and pregnancy outcomes, it remains an important avenue for future investigation. However, our novel analyses relating the *AGPome* and *CRPome* to various morbidity outcomes are more proximal than pregnancy outcomes and, potentially, more biologically plausible to interpret. 

## 5. Conclusions

This study represents the first attempt to apply large-scale plasma proteomics in a typical rural South Asian population. The *AGPome* and *CRPome* included well-known inflammatory proteins, complement proteins, proteins involved in intracellular immunity, pregnancy-related anabolic proteins, and transporters of micronutrients. Our findings highlight composites of highly complex physiological processes of early pregnancy to reveal the breadth and complexity of early gestational inflammation associated with concentrations of AGP and CRP, which remain inapparent when only these two classic biomarkers are measured. 

These findings and those in a Nepalese child population [21] merit caution in interpretation when adjusting biochemical measures for these two biomarkers in population-based studies of nutrition and health [8]. Our results call for a need to deeply explore the diversity and regulation of inflammation to identify reliable and potentially more informative biomarkers of maternal health and its relation to nutritional status [67] and pregnancy outcomes that may be of public health utility.

## Figures and Tables

**Table 1:** Characteristics of 1st trimester pregnant women in the JiVitA-3 trial by participation status in the proteomics study ^1^.

	Proteomics Substudy (N = 435)	Not Selected (N = 973)
**Randomized supplement allocation**		
Multiple Micronutrients	210 (48.3%)	470 (48.3%)
Iron–Folic Acid	225 (51.7%)	503 (51.7%)
**Education**		
No schooling	100 (23.0%)	258 (26.5%)
Grade 1 to Grade 4	65 (14.9%)	116 (11.9%)
Grade 5 to Grade 9	240 (55.2%)	509 (52.3%)
10 years and above	30 (6.9%)	90 (9.3%)
**Age (years)**		
<20	147 (33.8%)	304 (31.2%)
20–29	240 (55.2%)	546 (56.1%)
≥30	48 (11.0%)	123 (12.6%)
**Parity**		
0	167 (38.4%)	384 (39.4%)
1	139 (31.9%)	309 (31.8%)
≥2	129 (29.6%)	280 (28.8%)
**Gestational age at blood draw (wk), median (IQR)**	9.7 (8.0, 12.3)	10.1 (8.3, 13.4)
**Maternal anthropometry, mean (SD)**		
Height, cm	149.24 (5.18)	149.0 (5.3)
Weight, kg,	42.37 (5.76)	42.9 (6.2)
BMI, kg/m^2^	18.99 (2.11)	19.3 (2.3)
**Maternal malnutrition**		
BMI < 18.5 kg/m^2^	194 (44.6%)	398 (41.0%)
Height < 150 cm, %	228 (52.4%)	547 (56.3%)
Mid-upper arm circumference <21.5 cm, %	72 (16.6%)	178 (18.3%)
Living standard index, median (IQR)	−0.22 (−0.70, 0.43)	−0.20 (−0.70, 0.67)
**Laboratory values**		
α-1 acid glycoprotein, g/L	0.81 (0.30)	0.73 (0.28)
C-reactive protein, mg/L^2^	2.24 (5.25)	NA
α-1 acid glycoprotein >1 g/L	85 (19.5)	138 (14.2)
C-reactive protein >5 mg/L, % ^2^	43 (11.4)	NA
**Morbidity symptoms (days/wk, yes, %)**		
Nausea	224 (51.5%)	446 (45.8%)
Vomiting	117 (26.9%)	237 (24.4%)
Severe headache	31 (7.1%)	80 (8.2%)
High fever	6 (1.4%)	20 (2.1%)
Low fever	133 (30.6%)	310 (31.9%)
Diarrhea	2 (0.5%)	7 (0.7%)

^1^ Data are summarized as numbers and percentages (%) unless otherwise noted. SD = standard deviation; IQR = interquartile range; NA = not available. ^2^ A total of 15 women had missing data on C-reactive protein, providing an N = 420 for this analysis. NA, not applicable because these data were not collected.

**Table 2:** Top 50 proteins correlated with α-1 acid glycoprotein (AGP) *.

Entrez Gene Symbol	Protein Full Name	Entrez Gene ID	UniProt ID	Pearson *r*	Abs Δ/2x Protein	Standard Error	*p* Value	Q Value (FDR)
SERPINA4	Kallistatin ^#‡^	5267	P29622	−0.402	−0.630	0.069	2.49 × 10^−18^	1.89 × 10^−14^
APCS	Serum amyloid P-component ^#^	325	P02743	0.374	0.416	0.050	7.31 × 10^−16^	1.85 × 10^−12^
C4BPA	C4b-binding protein alpha chain ^#^	722	P04003	0.342	0.398	0.053	2.38 × 10^−13^	4.53 × 10^−10^
SAA1	Serum amyloid A-1 protein ^#‡^	6288	P0DJI8	0.334	0.077	0.010	9.14 × 10^−13^	1.39 × 10^−9^
SAA2	Serum amyloid A-2 protein ^#^	6289	P0DJI9	0.307	0.164	0.024	6.23 × 10^−11^	7.89 × 10^−8^
ARL5B	ADP-ribosylation factor-like protein 5B ^#^	221079	Q96KC2	0.302	0.433	0.066	1.27 × 10^−10^	1.38 × 10^−7^
C9	Complement component C9 ^#‡^	735	P02748	0.289	0.376	0.060	8.37 × 10^−10^	7.95 × 10^−7^
AFM	Afamin ^#‡^	173	P43652	−0.288	−0.354	0.057	9.88 × 10^−10^	8.34 × 10^−7^
CCN5	WNT1-inducible-signaling pathway protein 2 ^#^	8839	O76076	−0.281	−0.188	0.031	2.31 × 10^−9^	1.75 × 10^−6^
RBP4	Retinol-binding protein 4 ^‡^	5950	P02753	−0.276	−0.292	0.049	4.94 × 10^−9^	3.41 × 10^−6^
TFF3	Trefoil factor 3 ^#‡^	7033	Q07654	−0.273	−0.074	0.013	6.85 × 10^−9^	4.34 × 10^−6^
SHBG	Sex hormone-binding globulin ^#‡^	6462	P04278	−0.272	−0.106	0.018	8.43 × 10^−9^	4.93 × 10^−6^
CRP	C-reactive protein ^#^	1401	P02741	0.270	0.051	0.009	1.04 × 10^−8^	5.65 × 10^−6^
ITIH2	Inter-alpha-trypsin inhibitor heavy chain H2 ^#^	3698	P19823	−0.268	−0.424	0.073	1.45 × 10^−8^	6.89 × 10^−6^
HSPA1A	Heat shock 70 kDa protein 1A ^#^	3303	P0DMV8	−0.263	−0.275	0.049	2.54 × 10^−8^	1.13 × 10^−5^
AHSG	Alpha-2-HS-glycoprotein	197	P02765	−0.259	−0.359	0.064	4.43 × 10^−8^	1.87 × 10^−5^
C1QC	Complement C1q subcomponent subunit C ^#^	714	P02747	0.250	0.098	0.018	1.21 × 10^−7^	4.16 × 10^−5^
CFB	Complement factor B ^#^	629	P00751	0.251	0.403	0.075	1.17 × 10^−7^	4.16 × 10^−5^
ITIH5	Inter-alpha-trypsin inhibitor heavy chain H5	80760	Q86UX2	−0.251	−0.141	0.026	1.19 × 10^−7^	4.16 × 10^−5^
CFI	Complement factor I ^#^	3426	P05156	0.246	0.513	0.097	1.96 × 10^−7^	6.49 × 10^−5^
CEP76	Centrosomal protein of 76 kDa	79959	Q8TAP6	0.245	0.229	0.044	2.21 × 10^−7^	7.01 × 10^−5^
BTD	Biotinidase ^#^	686	P43251	−0.244	−0.372	0.071	2.44 × 10^−7^	7.42 × 10^−5^
MFAP2	Microfibrillar-associated protein 2	4237	P55001	−0.235	−0.246	0.049	7.32 × 10^−7^	2.06 × 10^−4^
LIPG	Endothelial cell-derived lipase ^#^	9388	Q9Y5X9	0.234	0.217	0.043	7.73 × 10^−7^	2.10 × 10^−4^
SERPINA11	Serpin A11	256394	Q86U17	−0.224	−0.171	0.036	2.39 × 10^−6^	5.86 × 10^−4^
HYAL1	Hyaluronidase-1	3373	Q12794	−0.221	−0.200	0.042	3.10 × 10^−6^	7.13 × 10^−4^
FBLN7	Fibulin-7	129804	Q53RD9	−0.219	−0.186	0.040	4.13 × 10^−6^	9.23 × 10^−4^
DDR1	Epithelial discoidin domain-containing receptor 1	780	Q08345	−0.218	−0.176	0.038	4.64 × 10^−6^	9.52 × 10^−4^
SLC5A8	Sodium-coupled monocarboxylate transporter 1	160728	Q8N695	−0.218	−0.186	0.040	4.52 × 10^−6^	9.52 × 10^−4^
IGSF3	Immunoglobulin superfamily member 3	3321	O75054	−0.218	−0.121	0.026	4.44 × 10^−6^	9.52 × 10^−4^
SERPINA7	Thyroxine-binding globulin ^#^	6906	P05543	−0.216	−0.340	0.074	5.22 × 10^−6^	1.04 × 10^−3^
IFIT3	Interferon-induced protein with tetratricopeptide repeats 3	3437	O14879	0.215	0.088	0.019	5.92 × 10^−6^	1.15 × 10^−3^
PEF1	Peflin	553115	Q9UBV8	−0.214	−0.242	0.053	6.53 × 10^−6^	1.24 × 10^−3^
TMEM132A	Transmembrane protein 132A	54972	Q24JP5	−0.214	−0.115	0.025	6.69 × 10^−6^	1.24 × 10^−3^
F11	Coagulation Factor XI	2160	P03951	0.213	0.215	0.048	7.60 × 10^−6^	1.35 × 10^−3^
SERPINA3	Alpha-1-antichymotrypsin complex	12	P01011	0.210	0.121	0.027	1.05 × 10^−5^	1.82 × 10^−3^
MLYCD	Malonyl-CoA decarboxylase, mitochondrial	23417	O95822	0.208	0.353	0.080	1.17 × 10^−5^	1.89 × 10^−3^
TLR5	Toll-like receptor 5	7100	O60602	0.208	0.223	0.050	1.17 × 10^−5^	1.89 × 10^−3^
AGT	Angiotensinogen ^#^	183	P01019	−0.209	−0.107	0.024	1.16 × 10^−5^	1.89 × 10^−3^
MX1	Interferon-induced GTP-binding protein Mx1	4599	P20591	0.208	0.062	0.014	1.19 × 10^−5^	1.89 × 10^−3^
ISG15	Ubiquitin-like protein ISG15	9636	P05161	0.207	0.116	0.026	1.35 × 10^−5^	2.09 × 10^−3^
SELPLG	P-selectin glycoprotein ligand 1 ^#^	6404	Q14242	0.200	0.247	0.058	2.55 × 10^−5^	3.80 × 10^−3^
PLA2G7	Platelet-activating factor acetylhydrolase	7941	Q13093	−0.198	−0.139	0.033	3.13 × 10^−5^	4.49 × 10^−3^
C1QL2	Complement C1q-like protein 2	165257	Q7Z5L3	−0.198	−0.191	0.045	3.11 × 10^−5^	4.49 × 10^−3^
SERPING1	Plasma protease C1 inhibitor	710	P05155	0.197	0.182	0.043	3.37 × 10^−5^	4.74 × 10^−3^
PCYOX1	Prenylcysteine oxidase 1	51449	Q9UHG3	−0.197	−0.167	0.040	3.46 × 10^−5^	4.78 × 10^−3^
APOA1	Apolipoprotein A-I	335	P02647	−0.196	−0.143	0.034	3.71 × 10^−5^	5.04 × 10^−3^
PGRMC2	Membrane-associated progesterone receptor component 2	10424	O15173	−0.196	−0.077	0.019	3.78 × 10^−5^	5.04 × 10^−3^
BGN	Biglycan	633	P21810	−0.195	−0.100	0.024	4.08 × 10^−5^	5.34 × 10^−3^
HS6ST1	Heparan-sulfate 6-O-sulfotransferase 1	9394	O60243	−0.195	−0.246	0.060	4.35 × 10^−5^	5.60 × 10^−3^

* Simple linear regression models were used to correlate relative plasma abundance of 6431 proteins with per doubling of AGP. Top 50 proteins with smallest Q values (FDR) are presented. ^#^ Indicates proteins that overlap between AGP and CRP. ^‡^ Indicates proteins with duplicate aptamers. Table includes aptamers with stronger association with AGP. Supplemental Tables S1 and S2 show full list of protein targets that are correlated with AGP at Q values (FDR) < 0.05. FDR, false discovery rate.

**Table 3:** Top 50 proteins correlated with CRP *.

Entrez Gene Symbol	Protein Full Name	Entrez Gene ID	UniProt ID	Pearson *r*	Percent Δ/2x Protein	Standard Error	*p* Value	Q Value (FDR)
CRP	C-reactive protein ^#^	1401	P02741	0.894	1.190	0.029	1.16 × 10^−147^	8.48 × 10^−144^
SAA1	Serum amyloid A-1 protein ^#‡^	6288	P0DJI8	0.654	1.067	0.060	1.57 × 10^−52^	5.72 × 10^−49^
SAA2	Serum amyloid A-2 protein ^#^	6289	P0DJI9	0.556	2.086	0.152	1.65 × 10^−35^	4.02 × 10^−32^
CLPSL1	Colipase-like protein 1	340204	A2RUU4	0.544	2.878	0.217	9.92 × 10^−34^	1.81 × 10^−30^
TXNL4A	Thioredoxin-like protein 4A	10907	P83876	0.524	2.140	0.170	5.09 × 10^−31^	7.42 × 10^−28^
LBP	Lipopolysaccharide-binding protein ^#^	3929	P18428	0.515	2.052	0.167	7.85 × 10^−30^	9.54 × 10^−27^
KIF3A	Kinesin-like protein KIF3A ^#^	11127	Q9Y496	0.511	4.759	0.391	2.24 × 10^−29^	2.34 × 10^−26^
C9	Complement component C9 ^#‡^	735	P02748	0.480	4.432	0.396	1.39 × 10^−25^	1.26 × 10^−22^
APCS	Serum amyloid P-component ^#^	325	P02743	0.469	3.667	0.338	2.33 × 10^−24^	1.89 × 10^−21^
SULT2B1	Sulfotransferase family cytosolic 2B member 1 ^#^	6820	O00204	0.445	4.480	0.441	8.35 × 10^−22^	6.08 × 10^−19^
PGLYRP2	N-acetylmuramoyl-L-alanine amidase	114770	Q96PD5	−0.418	−3.219	0.342	3.24 × 10^−19^	2.15 × 10^−16^
ALB	Serum albumin ^#^	213	P02768	−0.417	−4.118	0.439	4.48 × 10^−19^	2.51 × 10^−16^
CFB	Complement factor B ^#^	629	P00751	0.417	4.766	0.508	4.35 × 10^−19^	2.51 × 10^−16^
PLA2G2A	Phospholipase A2, membrane associated ^#^	5320	P14555	0.409	2.242	0.245	2.47 × 10^−18^	1.29 × 10^−15^
SAA1	Serum amyloid A-1 protein ^#^	6288	P0DJI8	0.404	0.597	0.066	6.30 × 10^−18^	3.06 × 10^−15^
SERPINA4	Kallistatin ^#‡^	5267	P29622	−0.401	−4.030	0.450	1.10 × 10^−17^	5.01 × 10^−15^
LRIG1	Leucine-rich repeats and immunoglobulin-like domains protein 1	26018	Q96JA1	0.395	2.410	0.274	4.14 × 10^−17^	1.78 × 10^−14^
CLEC3B	Tetranectin	7123	P05452	−0.389	−3.706	0.430	1.32 × 10^−16^	5.35 × 10^−14^
CFI	Complement factor I ^#^	3426	P05156	0.371	5.489	0.672	3.68 × 10^−15^	1.34 × 10^−12^
LIPG	Endothelial cell-derived lipase ^#^	9388	Q9Y5X9	0.368	2.414	0.298	6.68 × 10^−15^	2.21 × 10^−12^
FGL1	Fibrinogen-like protein 1	2267	Q08830	0.364	1.302	0.163	1.33 × 10^−14^	4.21 × 10^−12^
LSAMP	Limbic system-associated membrane protein	4045	Q13449	−0.357	−3.273	0.418	4.25 × 10^−14^	1.29 × 10^−11^
HTRA1	Serine protease HTRA1	5654	Q92743	0.345	2.054	0.273	3.46 × 10^−13^	1.01 × 10^−10^
GDF11|MSTN	Growth/differentiation factor 11/8	10220|2660	O95390|O14793	−0.343	−2.122	0.284	4.68 × 10^−13^	1.31 × 10^−10^
F9	Coagulation factor IX	2158	P00740	0.340	3.971	0.537	7.50 × 10^−13^	2.02 × 10^−10^
TNXB	Tenascin-X	7148	P22105	−0.334	−2.088	0.288	2.05 × 10^−12^	5.34 × 10^−10^
PLBD1	Phospholipase B-like 1	79887	Q6P4A8	0.332	2.013	0.280	2.77 × 10^−12^	6.95 × 10^−10^
AOC3	Vascular adhesion protein-1	8639	Q16853	−0.328	−2.235	0.315	5.76 × 10^−12^	1.40 × 10^−9^
FGFR1	Fibroblast growth factor receptor 1	2260	P11362	−0.323	−2.647	0.379	1.17 × 10^−11^	2.74 × 10^−9^
LUM	Lumican	4060	P51884	−0.322	−2.523	0.363	1.42 × 10^−11^	3.24 × 10^−9^
RET	Proto-oncogene tyrosine-protein kinase receptor Ret	5979	P07949	0.321	0.997	0.144	1.54 × 10^−11^	3.40 × 10^−9^
CFHR5	Complement factor H-related protein 5 ^‡^	81494	Q9BXR6	0.319	1.558	0.227	2.31 × 10^−11^	4.95 × 10^−9^
NPW	Neuropeptide W	283869	Q8N729	−0.318	−0.777	0.113	2.38 × 10^−11^	4.95 × 10^−9^
NTM	Neurotrimin ^‡^	50863	Q9P121	−0.318	−2.093	0.305	2.46 × 10^−11^	4.97 × 10^−9^
NEO1	Neogenin	4756	Q92859	−0.318	−2.476	0.361	2.53 × 10^−11^	4.99 × 10^−9^
CNTN1	Contactin-1	1272	Q12860	−0.312	−2.277	0.340	6.60 × 10^−11^	1.23 × 10^−8^
ALDH7A1	Alpha-aminoadipic semialdehyde dehydrogenase	501	P49419	0.310	1.588	0.238	8.09 × 10^−11^	1.47 × 10^−8^
SPON1	Spondin-1	10418	Q9HCB6	−0.309	−2.860	0.430	9.20 × 10^−11^	1.64 × 10^−8^
CHAD	Chondroadherin	1101	O15335	−0.309	−1.115	0.168	1.00 × 10^−10^	1.73 × 10^−8^
CFHR1	Complement factor H-related protein 1	3078	Q03591	0.305	3.337	0.510	1.72 × 10^−10^	2.84 × 10^−8^
APOA5	Apolipoprotein A-V	116519	Q6Q788	0.304	1.032	0.158	1.89 × 10^−10^	3.00 × 10^−8^
HS6ST3	Heparan-sulfate 6-O-sulfotransferase 3	266722	Q8IZP7	−0.304	−2.697	0.413	1.90 × 10^−10^	3.00 × 10^−8^
TP53	Cellular tumor antigen p53 R175H mutant	7157	P04637	0.302	2.851	0.440	2.61 × 10^−10^	4.04 × 10^−8^
C2	Complement C2	717	P06681	0.301	3.094	0.479	2.99 × 10^−10^	4.54 × 10^−8^
CHL1	Neural cell adhesion molecule L1-like protein	10752	O00533	−0.300	−2.903	0.451	3.27 × 10^−10^	4.87 × 10^−8^
CFHR5	Complement factor H-related protein 5	81494	Q9BXR6	0.300	1.707	0.265	3.39 × 10^−10^	4.94 × 10^−8^
TFF3	Trefoil factor 3	7033	Q07654	0.297	0.660	0.104	5.04 × 10^−10^	7.21 × 10^−8^
CHL1	Neural cell adhesion molecule L1-like protein	10752	O00533	−0.296	−1.841	0.291	6.08 × 10^−10^	8.52 × 10^−8^
LILRA5	Leukocyte immunoglobulin-like receptor subfamily A member 5	353514	A6NI73	0.296	2.505	0.396	6.30 × 10^−10^	8.66 × 10^−8^

* Simple linear regression models were used to correlate relative plasma abundance of 6431 proteins with per doubling of CRP. Top 50 proteins with smallest Q values (FDR) are presented. ^#^ Indicates proteins that overlap between AGP and CRP. CRP was log_2_-transformed. ^‡^ Indicates proteins with duplicate aptamers. Table includes aptamers with stronger association with CRP. Supplemental Tables S3 and S4 show full list of protein targets that are correlated with AGP at Q values (FDR) < 0.05. FDR, false discovery rate.

**Table 4:** Odds of reporting maternal morbidity symptoms per 1 standard deviation (SD) higher in principal component scores *.

		Symptoms Reported in the Past 7 Days					
		Nausea	Vomiting	Severe Headache	High Fever	Low Fever	Diarrhea
Number of participants reporting symptoms (yes, %)		224 (51.5)	117 (26.9)	31 (7.1)	6 (1.4)	133 (30.6)	2 (0.5)
α-1 acid glycoprotein (AGP) (n = 147 proteins)							
	PC1 (22% variance explained)	1.04 (0.97, 1.12)	**1.15 (1.04, 1.28)**	1.17 (0.97, 1.41)	**2.28 (1.59, 3.29)**	1.08 (0.99, 1.17)	0.66 (0.15, 2.10)
	PC2 (9% variance explained)	1.00 (0.93, 1.08)	1.05 (0.95, 1.17)	**1.22 (1.01, 1.48)**	**2.05 (1.37, 3.11)**	1.06 (0.97, 1.15)	0.60 (0.18, 1.89)
	PC3 (6% variance explained)	**1.29 (1.19, 1.39)**	**1.13 (1.01, 1.26)**	0.98 (0.82, 1.19)	**1.69 (1.08, 2.77)**	0.98 (0.90, 1.07)	0.68 (0.31, 2.01)
C-reactive protein (CRP) (n = 879 proteins)							
	PC1 (24% variance explained)	0.97 (0.90, 1.04)	0.94 (0.85, 1.04)	1.08 (0.89, 1.31)	1.15 (0.79, 1.70)	1.01 (0.92, 1.09)	0.91 (0.28, 2.89)
	PC2 (9% variance explained)	0.97 (0.91, 1.05)	1.06 (0.95, 1.17)	**1.22 (1.02, 1.45)**	0.96 (0.64, 1.39)	1.07 (0.99, 1.17)	0.59 (0.13, 1.91)
	PC3 (5% variance explained)	1.05 (0.97, 1.12)	**1.19 (1.07, 1.33)**	1.05 (0.87, 1.28)	**2.52 (1.54, 4.33)**	1.01 (0.93, 1.10)	0.76 (0.27, 2.47)

* We performed principal component analysis (PCA) and explored linear combinations of proteins that were significantly associated with AGP and CRP. Then, we linked these PCA with a positive 7-day history of maternal morbidity symptoms. Statistically significant results were **bolded**.

## Data Availability

Data from the present study will not be shared publicly and will follow the parent study’s (JiVitA trial) data management and sharing procedures.

## Supplementary Materials

The following supporting information can be downloaded at https://www.mdpi.com/article/10.3390/biom14070736/s1, Table S1: Proteins positively correlated with AGP at FDR < 0.05; Table S2: Proteins negatively correlated with AGP at FDR < 0.05; Table S3: Proteins positively correlated with CRP at FDR < 0.05; Table S4: Proteins negatively correlated with CRP at FDR < 0.05; Table S5: Factor loadings from principal component analysis of plasma proteins significantly associated with α-1-acid glycoprotein in 435 Bangladeshi pregnant women; Table S6: Factor loadings from principal component analysis of 879 plasma proteins significantly associated with c-reactive protein glycoprotein in 435 Bangladeshi pregnant women.
